# Promoting patient rights in nursing care in Ghana through the Caring Space Model

**DOI:** 10.4314/gmj.v59i3.9

**Published:** 2025-09

**Authors:** Abukari Kwame

**Affiliations:** 1 College of Nursing, University of Saskatchewan, Prince Albert Campus, SK, Canada

**Keywords:** caring space, person-centred care, patient rights, patients' charter, patient education, Ghana

## Abstract

**Objectives:**

To explore patient rights outcomes in nurse-patient clinical interactions in the Yendi Hospital and promote patients' rights in Ghana using a proposed Caring Space Model.

**Design:**

An ethnographic research design was implemented, and purposive sampling was used to recruit participants. Data were gathered across nine inpatient units through in-depth individual interviews (n = 39), ethnographic participant observations (over 400 hours), and a focus group discussion from December 2021 to April 2022. A reflexive thematic analysis was conducted to explore participants' knowledge, experiences, and barriers to upholding patient rights in clinical interactions.

**Setting:**

The study was conducted in the Yendi Municipal Hospital.

**Participants:**

Included Nurses (n=11), patients (n=21), and caregivers (n=11) who were 18 years of age or older and provided voluntary consent. Additionally, nurses must have at least three years of experience in a hospital setting.

**Results:**

The study found that most patients and caregivers are unaware of the Ghanaian *Patients' Charter*. Nurses who were familiar with the *Charter* failed to educate patients and caregivers because they feared that doing so would cause undue stress. Poor patient rights outcomes were rooted in human and material resource constraints, affecting nurse-patient and nurse-nurse manager relationships. Patient rights education, transformative nursing leadership practices, and effective communication during clinical interactions can enhance patient rights and safety.

**Conclusion:**

To promote patient-centred care, a model of the Caring Space has been developed to advance ethical nursing and caring practices that elevate patient rights in patient-provider clinical interactions.

**Funding:**

**No funding**

## Introduction

Promoting patient rights is critical to realising positive person-centred care (PCC) outcomes. According to the European Declaration on the Rights of Patients, patient rights are fundamental human rights in healthcare, aimed at protecting patients' dignity and integrity, and respecting the patient as a person (World Health Organisation [WHO]).^1^ Both the International Council of Nurses (ICN)^2^ and WHO^3^ emphasise that respecting patient rights is essential for improving quality of care, ensuring patient safety, and fostering active participation in the healthcare process.

The right to health and patient rights are collectively referred to as ‘human rights in patient care.’^4^ These rights are critical for effective care delivery and positive provider-patient interactions because upholding patient rights is crucial to achieving Sustainable Development Goal (SDG3),^5^,^6^ because patients' access to available and affordable healthcare services and facilities can be constrained if healthcare providers dehumanise and maltreat patients and their caregivers.

Similarly, nursing ethics and standards of practice are important for delivering efficient nursing care, as they enable healthcare providers to meet the healthcare needs of patients and caregivers.^2^ In particular, the ICN ethical codes require nurses to fulfil four essential responsibilities: promoting health, preventing illness, restoring health, and alleviating suffering.^2^,^7^ As such, nursing ethical codes guarantee that “inherent in nursing is a respect for human rights, including cultural rights, the right to life, choice, dignity, and to be treated with respect.”^7^ Thus, nurses have a professional responsibility to provide care for people requiring it while promoting “an environment in which the human rights, values, customs, and spiritual beliefs of patients and caregivers are respected.”^7^

Therefore, promoting patient rights is inherent in nursing philosophy and practice, and non-negotiable when providing care to patients.

Despite the central position that patient rights occupy in nursing care and practice, studies in Ghana have shown that patient rights are often disregarded.^8^,^9^,^10^ Zutah et al.^11^ explored medical misconduct among healthcare professionals in Ghana by analysing legal cases, news reports, and other empirical studies. Findings revealed that medical malpractices, patient negligence, breach of duty of care, and failure in healthcare institutional oversight and security responsibilities are commonplace.^11^ Similarly, Yarney et al.^10^ observed that violations of patients' rights are prevalent in Ghana's healthcare institutions despite the *Patients' Charter*, which was established to promote patients' rights and responsibilities.

This paper examines the knowledge and experiences of patients, nurses, and caregivers regarding patient rights, as well as the barriers to promoting these rights at Yendi Hospital. Two questions are addressed: (a) How do patients, caregivers, and nurses perceive patient rights? and (b) How can these participants' experiences of patient rights inform rights-based healthcare practice? Additionally, the paper presents a model, known as the Caring Space, and its potential to foster rights-based care practices and interactions among patients, caregivers, and healthcare professionals in Ghanaian healthcare institutions.

### The Ghanaian Patients' Charter

In 2002, the Ministry of Health and Ghana Health Service (GHS) drafted and approved the *Patients' Charter* for use in Ghana.^12^ The *Charter* has 4 General Articles (a, b, c, d) stressing the need for quality care, patients' engagement in decision-making, protection against discrimination, and health promotion. It also contains 14 unique patient rights provisions addressing quality care (*Article* 1), information (on healthcare cost, procedures, alternatives, and healthcare provider identity) (*Articles* 2, 3, 4, 9, 10, 11, 12, 14), patient consent (including that for research purposes, *Articles* 5, 6), privacy and confidentiality (*Articles* 7, 8), and patient safety (*Article* 13).^12^ Furthermore, the *Charter* provides nine responsibilities of patients/caregivers, including providing/requesting information (Obligations 1, 2, 4, 5), treatment compliance (Obligation 3), health education/knowledge acquisition (Obligation 6), promoting a safe environment (Obligation 7), respecting others' rights (Obligation 8), and protecting healthcare facility's property (Obligation 9).^12^ The *Charter* declares, “In all healthcare activities, the patient's dignity and interest must be paramount.”^12^

Similarly, the *Nursing Code of Ethics* and the revised *Code of Conduct and Disciplinary Procedures* for healthcare professionals^13^ further support patient rights by emphasizing the importance of observing these rights and professional codes of conduct in all healthcare facilities in Ghana. Lastly, patient rights provisions in the *Charter* are anchored in Article 30 (Rights of the Sick) of the 1992 Constitution of Ghana, which stipulates that a patient who is unable to provide consent due to ill health shall not be deprived of medical treatment, education, or any other benefit, be it social or economic, due to their religious or other beliefs.^14^ Again, many provisions under *Chapter 5* (Fundamental Human Rights and Freedoms) of the Constitution underscore Respect for Human Dignity and non-discrimination, as significant patient rights principles in Ghana.^14^

## Methods

### Research design

An exploratory qualitative research design was implemented in the doctoral research project, part of whose data are reported in this paper. Institutional ethnographic approaches^15^ were employed to examine everyday clinical practices and social interactions within the hospital and their impact on patient rights. Attention was paid to the everyday dynamics of clinical practices, patterns of interactions, and communication practices, as well as how the healthcare context and institutional culture shape interpersonal interactions and patient experiences of their rights in the hospital.^16^,^17^ Nurses', patients', and caregivers' lived experiences of patient rights in nurse-patient clinical interactions and how communication practices impacted these rights were explored.

### Study setting and participants

The study was conducted in a public hospital in Yendi, Ghana. Eleven nurses, 11 caregivers, and 21 patients were purposively sampled across nine inpatient wards. Purposive sampling allowed participants who were willing, available, and had experiences of patient rights to participate and share their perspectives.^18^ Participants were invited through word of mouth and a recruitment poster. The hospital gatekeepers and a few nurses facilitated the process. Participants provided their informed consent by signing or thumbprinting the consent forms.

### Data collection and analysis

Semi-structured individual interviews (n=39) were conducted with all nurses, caregivers, and 17 patients to understand their lived experiences.^19^,^20^ One focus group (with four patients) was held, and over 400 hours of ethnographic participant observations were conducted across the patient wards and other spaces within the hospital. I had informal conversations with nurses at the nurses' stations and formal meetings with specific hospital officials to gain additional data. Interviews were conducted in Dagbani among four patients and caregivers, and in English with all nurses, patients, and caregivers. These interviews were audio recorded and later transcribed verbatim (for interviews in English) or translated directly into English while retaining crucial Dagbani concepts and expressions for interviews conducted in Dagbani (a language I am a native speaker of).

All data transcripts, including typed field notes, were manually coded inductively and analysed thematically using Braun and Clarke's^21^,^22^ reflexive thematic analysis. For details about data collection and analysis, see Kwame.^23^ Also, I analysed the Ghanaian *Patient's Charter* document to determine how particular nurse-patient-caregiver interactional episodes and communicative practices aligned with or violated the *Charter* provisions. My engagement with the doctoral committee, presenting the preliminary findings at seminars, and also sharing that with the hospital community helped fine-tune my themes and interpretive analysis. Ethical approvals were granted by the University of Saskatchewan Behavioural Ethics Committee (Beh-ID: 2690), the Ghana Health Service (GHS-ERC:005/11/21), and the Yendi Hospital.

## Results

The themes reported in this paper capture participants' experiences of patient rights in the Yendi Hospital, and drawing on their understanding of care and caring nursing practices, a model was developed to promote rights-conforming care in Ghana.

### Participants' demographics

A total of 43 participants, consisting of 27 females and 16 males, participated in the study. Many of the participants were Dagomba. The youngest participant was 18 years old, while the oldest was 60. Among patient participants, the average length of hospital stay was three days, with the longest stay being 2 weeks. Nurse participants had an average of six years of practice experience in the hospital. Except for a few participants, most were bilingual, speaking more than one Ghanaian language in addition to English. The demographic characteristics of the participants are shown in Table 1.

**Table 1:** Participants' demographic characteristics

Characteristics	Patients	Caregivers	Nurses
Sample size	21	11	11
Marital status			
Married	9	8	9
Single	12	3	2
Age (in years)			
Range	18 – 60	19 – 45	26 – 40
Mean age	26	32	33
Participants below the mean	13	6	6
Sex			
Male	5	4	7
Female	16	7	4
Ethnicity			
Dagomba	13	8	8
Ewe	2	2	0
Konkomba	2	0	0
Others	4	1	3
Level of Education			
None	3	2	0
Basic	2	6	0
Secondary	10	1	0
Tertiary	6	2	11
Occupation			
Nursing	0	0	11
Student	7	1	0
Trading	4	2	0
Farming	3	6	0
Teaching	3	1	0
Other	4	1	0
Knowledge of the *Patient's Charter*			
Yes	3	0	9
No	18	11	2

### Knowledge of patient rights

Negative experiences of patient rights were partly due to the lack of knowledge about the *Patient's Charter* and its content among patients. Over 67% of the patients and caregivers (all caregivers and 41.9% of the remaining 18 patients) reported lacking knowledge about their rights and the *Charter*.

1. *I don't know about the Patients' Charter. (CG3)*
2. *The unfortunate thing is that patients don't know their rights. (N5)*.

Comparatively, nine nurses were aware of the *Patient's Charter* and even knew specific provisions of the *Charter*, as reported:

3. *The patient has the right to refuse treatment (N1). The patient has the right to privacy and confidentiality (N2). The right to know their conditions (N6), and Patients have the responsibility to take their medication. (N5)*

Only two nurses were unaware of the Charter or its content.

4. *I am aware of the Charter, but I can't remember the content (N3). Yes, I heard about the Charter, but I don't know much about it” (N8)*.

The above data suggest that patients' lack of knowledge about the *Charter* and its content limited their ability to influence healthcare providers to observe these rights. Furthermore, some nurses believe that nursing and caring activities will become challenging when patients and caregivers are enlightened about their rights. This perception demotivated some nurses from educating patients about these rights or implementing them during care delivery.

### Nurses' perceptions of patient rights

Some nurses had negative perceptions of patient rights promotion and failed to educate patients about these rights, thus disregarding *Articles 9, 10, and 11* of the *Charter*. The following excerpts illustrate these perceptions.

5. *I don't usually tell my patients their rights/responsibilities. (N4)*
6. *We don't often tell them their rights. (N5)*

Other nurses indicated that patients would make their work difficult if they knew their rights. These nurses feared that patients and caregivers might demand more care than nurses could deliver.

7. *We don't tell patients their rights [because] we fear that if you tell them this or that is your right, it will affect the care process. (N3)*
8. *[The Charter] does not emphasize patient responsibilities, and it puts nurses at risk because it doesn't give any rights to the nurse [or] emphasize nurses' rights. (N9)*

These perceptions could have prevented some nurses from promoting patient rights or learning the *Patient Charter*.

### Experiences of patient rights among patients

Patients and caregivers reported experiencing inadequate and adverse outcomes related to patient rights during nurse-patient interactions. That is, *Articles a & b were disregarded*, as reports of verbal and emotional abuse, threats, and patient neglect were reported.

9. *Yes, I saw one nurse threatening a patient that he would walk away from him. The patient had a mental health illness. So, when the nurse was interacting with him, and things were not going well, the nurse became frustrated and threatened to leave the patient. (CG1)*
10. *I witnessed something between a nurse and one elderly patient. The patient was feeling hot and decided to sprinkle water on himself, and the way the nurse spoke to the patient, I wasn't happy. The way the nurse spoke to the man was not proper. The nurse asked the man what he did; if it were in his home, would he have done that? He told the patient to let his caregivers mop the floor. That incident didn't make me happy. (P15)*

The patients' rights to safety (*Article 13*), respect, and dignity (*Articles a & b*) were compromised in the above narratives. Moreover, patients were often uninformed about their health condition, which denied them the right to information about their health (*Articles 2, 3, 4*).

11. *In this hospital, it is a normal phenomenon for patients not to know their condition*… *even a patient might be taking a drug without knowing its [side] effects. (N9)*

However, some patients experienced positive outcomes related to their patient rights.

12. *Since I came to this ward, I have not seen any differences in how nurses treat patients. The nurses give both rich and poor patients the same attention. So, there are no differences in how patients are treated. (P2)*

On the other hand, nurses' experiences of patient rights were attributed to poor staffing, resource constraints, and limited space (a theme I will explore later). These factors made it difficult for nurses to promote patient privacy and confidentiality or ensure timely and quality care delivery, thereby compromising *Articles 1, 7, 8*, and *13* of the *Charter*.

13. *Here [in this hospital], it's not easy to implement patient rights because sometimes when you are talking to a patient, other patients are listening to your conversation. (N2)*
14. *Due to the poor nurse-patient ratio, a patient may need more attention, but looking at the number of patients we have, you cannot give that patient the needed attention, and not giving him that attention, s/he may think we have neglected them. (N4)*

The above narratives illustrated how staff shortage and limited space affected nurses' ability to deliver timely care as well as respect patient privacy and confidentiality.

### Barriers to patient rights realisation

In addition to staff shortage and limited ward space, language barriers created linguicism, affecting patients' ability to engage in their care processes. A patient lamented how language barriers affected his participation in the care process and right to information (*Articles a, b, c, 2*).

15. *I am worried that I can't talk directly with the nurse. I would be happy to have a nurse who speaks my language so I can talk directly to him. (P4)*

Similarly, nurses complained about the impact of language barriers on their relationships with patients and caregivers, which led to a disregard for patient rights.

16. *Hmm, sometimes it's the language barrier. I don't understand the Konkomba language, and the neighbouring communities are Konkomba. So, it's just the language barrier; how to communicate with them is a big problem unless you find an interpreter. (N10)*

Thus, language barriers were a significant cause of patient rights violations, as participants indicated that misunderstandings often led to confrontations and quarrels due to miscommunication. Moreover, resource constraints impeded positive patient rights outcomes, including the right to prompt, safe, and quality care (*Articles a, 2, 13*).

17. *A motor accident patient brought to the emergency unit had his cuts stitched without a pain relief injection. At one point, the patient could no longer bear the pain. He told the nurses to stop with the stitching and pleaded with them to be patient with him. (Field notes, documented January 12, 2022)*

The above emergency patient's right to safe care was compromised. He was given several stitches without any pain relief injection, subjecting him to unbearable pain, which made him refuse further care.

Similarly, the lack of staff motivation affected patient rights and humane care practices among some nurses. Many nurses reported being unmotivated, which affected their passion for the job. Again, the lack of concern for nurses' welfare constrained their ability to deliver person-centred care or advocate for patient rights.

18. *We hear from the senior nurses that the hospital management doesn't help nurses get paid study leave, which shows the hospital doesn't care about us. So, yes, since the hospital doesn't care about me or my welfare, I will also do what I want in the ward, which may affect the patient. (N11)*

Therefore, language barriers, limited nurse staffing, lack of staff motivation and concern for nurses' welfare influenced nurses' care practices and willingness to align care with provisions of the *Patient's Charter*.

### The Caring Space Model

Drawing on participants' experiences of patient rights, their understanding of caring, and the characteristics of a caring nurse, the Caring Space model was developed from two critical themes: *Becoming a caring nurse* and *Honouring the ethics of life and caring*. Based on participants' responses to questions concerning who a caring nurse is, what caring nurses do, and how a nurse can become caring, specific attributes and values of caring that humanise patients and patient-provider interactions were identified. These values became the foundation of the Caring Space, designed to honour patient rights in Ghanaian healthcare facilities. When healthcare providers and their clients operate in the Caring Space, they can collectively promote patient rights and quality care.

#### Becoming a Caring Nurse

Participants stated that nurses can exhibit certain attributes during nurse-patient interactions that show whether they care for their patients. Nurses who demonstrated professionalism, provided timely care, communicated effectively, and respected patients' dignity were seen as caring.

19. *They [caring nurses] give listening ears to their patients. When a patient complains, they find out, and then if there is anything they can do to resolve the problem, they do it. They know how to care for their patients. (P14)*
20. *An uncaring nurse is always annoyed with patients or caregivers. Such a nurse doesn't even allow caregivers to see their patients, let alone interact with them. (CG8)*
21. *To be a caring nurse, you must put your patients first. It's because of them that I am here. So, they come first, and anything else comes after that. Caring nurses are punctual and responsive to patients' and caregivers' needs. (N9)*

Participants stated that respecting, tolerating, actively listening, and prioritising the patient and their needs in the care process make nurses caring, and that positive nurse-patient relationships are built when care providers demonstrate love and care. Caring elevates meaningful interactions between healthcare providers and patients/caregivers, leading to high-quality, right-conforming care.

#### Honour the Ethics of Life and Care

Participants further discussed what it means to be humane in the caring process by identifying values that promote care and respect for human life. They reported that being humane was the core ethics of life and care.

22. *As a nurse, you must realize that the client/patient you are dealing with is just as human as you are. Nurses are care providers; we give care and help patients to recover, and those who will not recover to die peacefully. (N8)*
23. *I am grateful to the nurses for their work because a human being is a human being; for any person to be sick in the hospital and have no caregiver and nobody here to support her is worrying. There is a patient in the ward, she has nobody with her. It is the nurses who care for and do everything for her. The nurses don't know her. Yet, they are caring for and supporting her. That is respecting life. (CG3)*
24. *I have just one message for nurses … they should know that they can also become patients one day. They should know that we are all humans. They have relatives like us, so they should treat patients as they would treat their relatives. (P5)*

**Figure 1:**
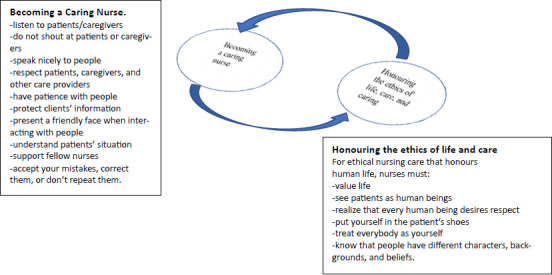
The Caring Space Mode The Caring Space connects caring values with honouring the ethics of life and care within patient-provider clinical interactions

These participants recognise that respect for life and humanity is the highest form of care (ethical care) one can receive in the hospital. As such, valuing life, respecting everyone, and recognising the uniqueness of all patients, caregivers, and providers were deemed the primary ethics of life and care. By combining the attributes of caring nurses with the principles of ethical care, a model of the Caring Space was developed, as depicted in Figure 1.

To be or become caring, healthcare professionals must honour the ethics of life and care that participants identified, allowing nursing and caring practices to flow organically. Healthcare professionals must place human life and well-being at the center of their professional practice. Additionally, caring nurses continually strive to improve their care by being open to learning from and with patients, and by supporting patients, caregivers, and their colleagues throughout the care delivery process. Therefore, to promote patient rights in the care delivery process, caring nurses must always be humane as they deliver care. Grounded in an African caring ethic, the participants underscored the value of humanness in nursing care, noting that “Our being human is always also a becoming human that is enabled through relating positively to others” in clinical interactions. 24

Therefore, the Caring Space invites care providers, patients, and patient families to embrace mutual respect and recognise one another's humanness as they relate and interact within that space.

## Discussion

This paper explored patient rights in a Ghanaian healthcare facility to understand the experiences of nurses, patients, and caregivers regarding patient rights during clinical interactions. The study found that enhancing patient rights education and awareness in Ghana is crucial because patients and caregivers lack knowledge about patient rights. The need for public awareness and education to increase Ghanaians' knowledge of the *Patients' Charter* is imperative, and recognised in the literature,^10^ yet the problem persists. Moreover, although the *Patient's Charter* is already included in nursing education in Ghana, it is not monitored during nursing students' clinical practice. Continuous education and training must be provided to nurses to build their capacity to promote patient rights through advocacy. The Commission on Human Rights and Administrative Justice (CHRAJ)^25^ previously organised training on patient and human rights for nurses and nursing students in Ghana to equip them with knowledge about patient rights and the *Charter*. This training opportunity must be extended to all nurses across Ghana.

Again, since many patients are less knowledgeable about their rights, the government, the Ministry of Health, the Ghana Health Service, the National Commission for Civic Education (NCCE), and CHRAJ should design programs to educate the public about the *Patient's Charter*.

Moreover, the study found that neglecting nurses' personal and professional needs, including motivating them, is a barrier to promoting patient rights. Nurses who feel unmotivated or whose nurse managers neglect their needs are less encouraged to promote or observe patient rights in care. Copies of the *Patient Charter* were distributed throughout the patient units in the hospital; however, some nurses were unaware of the Charter's content and did not educate patients about their rights. More visibility should be given to the *Patient's Charter* in patient wards, and patients should be informed about their rights during clinical interactions. Similarly, nurse burnout, limited staff, and resource constraints affected these nurses' emotional and psychological state, with adverse consequences for patients' right outcomes. Given the critical roles nurses play in care delivery, they must be motivated to enhance patient rights in Ghanaian healthcare facilities.

Furthermore, healthcare leaders must monitor patient rights outcomes in their institutions and support their staff in promoting these rights in practice by providing an enabling healthcare environment for nurses and other care providers to implement patient rights. Healthcare service providers, patients/clients, and caregivers should be empowered to understand their rights and responsibilities.^12^ Nevertheless, many hospitals may lack mechanisms for monitoring or enforcing provisions of the *Charter* in clinical practice. As a result, patient complaints, reports of abuses, and adverse rights outcomes must be documented, investigated, and resolved. Alternatively, legal scholars in Ghana have suggested that patient rights be enforced by creating a national healthcare law and an ombudsman to implement legal actions.^11^,^26^,^27^

Although the *Patients' Charter* prohibits discrimination based on culture, ethnicity, and language, it does not provide a guideline regarding how patients' and caregivers' language rights can be respected. Patients must have the right to receive healthcare in the language of their choice, given the impact of language barriers on access to care. This study found that nurses and patients face significant language barriers, which may be common in other Ghanaian hospitals. Therefore, there is an urgent need for a health language policy about nurses' linguistic capacity when posting them to serve in different communities. Posting nurses to communities where they are not linguistically capable of interacting with patients and their families violates patients' language rights, constituting a form of linguicism.^28^ Although trained translators and interpreters can help mitigate language barriers in hospitals, translating the *Charter* provisions into practice will require sensitivity to cultural differences in language use and notions of dignity in Ghana.

Additionally, healthcare quality control departments and officers must gather and evaluate data on human and patient rights practices within their institutions. In general, promoting patient rights should become a collective goal in healthcare facilities and institutions, and the *Caring Space* model can be a critical facilitation tool to enhance patient rights at the patient-provider level. Aligning care practices with this model honours what Roach^29^ regards as being in the caring mode. Nurses' perceptions and fears that informing patients about their rights will make their work more difficult must be abandoned. The ICN^2^ deemed patients' “right to dignity and to be treated with respect” as crucial. Thus, nurses must “promote an environment in which the human rights, values, customs, religious and spiritual beliefs of the individual, families and communities are acknowledged and respected.”^2^ As such, educating patients about their rights should be encouraged among all healthcare providers.

To achieve the *Patient's Charter* provisions, healthcare professionals will require more cultural competence training and education to position them well to interact with patients from diverse cultures in Ghana. This training will help them provide culturally sensitive care,^30^ respectful of patients' and caregivers' cultural values and worldviews on health and caring. The need for intercultural training for healthcare professionals in Ghana has been emphasised in the literature.^31^ Abdulai et al.^31^ recommended assessing “nurses' linguistic ability and cultural sensitivity” before posting them to the healthcare facilities they serve. Nursing training institutions must also include deliverable objectives as part of nursing students' clinical practice sessions to enable them to observe patient rights in clinical interactions as part of their clinical training.^23^

Lastly, communication should be emphasised in nursing education and practice to leverage positive nurse-patient interactions. When clinical communications are person-centred and respectful of the Caring Space model, all care practices can become rights-conforming, allowing patients, caregivers, and healthcare professionals to engage in respectful and meaningful conversations.

### Implications of the Caring Space Model for effective care delivery

The Caring Space model requires respecting human rights and patients' dignity at the core of nursing care. To honour life, one must value people regardless of their social status. Healthcare providers, patients, and caregivers must be tolerant and respectful of one another. The model encourages healthcare providers to treat patients and caregivers as they would treat themselves because disregard for patient rights often leads to maltreatment, poor interpersonal relationships, and hostile verbal exchanges between patients and healthcare providers in Ghana.^32^

This model demands nurses and other care providers to reflect on their professional behaviours and align their healthcare practices with ethical and caring values. They must honour the ethics of life and be humane to provide ethical care that eliminates interpersonal barriers to accessing care.^33^,^34^ To achieve that, the attributes of becoming caring must interlope with the values of honouring the ethics of life and care to create a Caring Space within which all patients and providers interact.

Again, the model recognises individual uniqueness, emphasising PCC, cultural sensitivity in clinical interactions, and positive therapeutic relationships, thereby invoking Peplau's Peplau's^35^-^37^ theory of Interpersonal Relations and the African Ubuntu philosophy and ethics of life,^38^ which makes it a resourceful tool in nursing practice. Peplau^35^-^37^ recognised that good relationships and ethical practices are significant in nurse-patient clinical interactions. Nurses are encouraged to respect patients' humanness, beliefs, and virtues, and position them as the focus of any communicative encounter. Similarly, Ubuntu is about valuing human well-being, respecting relationships, and embracing the ethics of life and what it means to be human.^24^,^38^, Following Seehawer's^24^ interpretation of the Ubuntu philosophy, I maintain that in all clinical spaces, everyone (including patients) must be engaged as a whole person in the care process, recognising the inherent power imbalances and vulnerabilities that exist between patients and healthcare providers.

Moreover, Gullick et al.^39^ observe that nurses who use their experiences and operate in a ‘self-mode,’ seeking connection [knowing] and openness [unknowing] with patients, will enhance positive nurse-patient relations and care outcomes compared to nurses who operate with a ‘they-mode’; where patients are seen as ‘diseased bodies’ that must be worked on to restore health. I invite all healthcare providers, patients, and caregivers to embrace mutual respect, realising that every life matters and deserves care with dignity.

## Conclusion

This study examined patient rights in patient-provider clinical interactions and demonstrates that advancing patient rights is vital to strengthening person-centred care and improving healthcare access in Ghana. Ensuring rights-based care requires widespread knowledge of the *Patient's Charter* among both providers and patients, active engagement of institutional leaders in rights-focused dialogues, and systematic efforts to reduce the impact of resource constraints and staff shortages. Incorporating human rights principles into healthcare language policy and applying the Caring Space model can further promote dignity, equity, and respectful interactions, thereby minimising conflict and discrimination in clinical practice.
